# The Deubiquitinase USP13 Maintains Cancer Cell Stemness by Promoting FASN Stability in Small Cell Lung Cancer

**DOI:** 10.3389/fonc.2022.899987

**Published:** 2022-07-11

**Authors:** Juhong Wang, Weihao Lin, Renda Li, Hong Cheng, Sijin Sun, Fei Shao, Yannan Yang, Lin Zhang, Xiaoli Feng, Shugeng Gao, Yibo Gao, Jie He

**Affiliations:** ^1^ Department of Thoracic Surgery, National Cancer Center/National Clinical Research Center for Cancer/Cancer Hospital, Chinese Academy of Medical Sciences and Peking Union Medical College, Beijing, China; ^2^ State Key Laboratory of Molecular Oncology, National Cancer Center/National Clinical Research Center for Cancer/Cancer Hospital, Chinese Academy of Medical Sciences and Peking Union Medical College, Beijing, China; ^3^ Laboratory of Translational Medicine, National Cancer Center/National Clinical Research Center for Cancer/Cancer Hospital, Chinese Academy of Medical Sciences and Peking Union Medical College, Beijing, China; ^4^ Department of Pathology, National Cancer Center/National Clinical Research Center for Cancer/Cancer Hospital, Chinese Academy of Medical Sciences and Peking Union Medical College, Beijing, China

**Keywords:** USP13, cancer stem cells, FASN, ubiquitylation, lipogenesis, TVB-2640, SCLC

## Abstract

*USP13* is significantly amplified in over 20% of lung cancer patients and critical for tumor progression. However, the functional role of USP13 in small cell lung cancer (SCLC) remains largely unclear. In this study, we found that the deubiquitinase USP13 is highly expressed in SCLC tumor samples and positively associated with poor prognosis in multiple cohorts. *In vitro* and *in vivo* depletion of USP13 inhibited SCLC cancer stem cells (CSCs) properties and tumorigenesis, and this inhibitory effect was rescued by reconstituted expression of wide type (WT) USP13 but not the enzyme-inactive USP13 mutant. Mechanistically, USP13 interacts with fatty acid synthase (FASN) and enhances FASN protein stability. FASN downregulation suppresses USP13-enhanced cell renewal regulator expression, sphere formation ability, and *de novo* fatty acids biogenesis. Accordingly, we found FASN expression is upregulated in surgical resected SCLC specimens, positively correlated with USP13, and associated with poor prognosis of SCLC patients. More importantly, the small molecule inhibitor of FASN, TVB-2640, significantly inhibits lipogenic phenotype and attenuates self-renewal ability, chemotherapy resistance and USP13-mediated tumorigenesis in SCLC. Thus, our study highlights a critical role of the USP13-FASN-lipogenesis axis in SCLC cancer stemness maintenance and tumor growth, and reveals a potential combination therapy for SCLC patients.

## Introduction

Small-cell lung cancer (SCLC) is a neuroendocrine (NE), early metastatic, rapidly progressing and therapy-resistant lung cancer, with 5-year survival of 15%–30% for limited-stage disease, and less than 1% for patients with extensive-stage disease according to the Veteran Affairs Lung Group staging criteria (1–3). Although the sequencing results have provided a better understanding of the signaling pathways in SCLC, most recurrent genetic events are not directly linked to obvious regulatory network. Recently, proteomic analyses revealed other putative vulnerabilities that eventually led to the identification of improved therapeutic strategies (4–6), suggesting the importance of identifying targetable proteins that directly contribute to tumor development or drug resistance. Cancer stem cells (CSCs) in SCLC play a central role in tumorigenesis, metastasis, drug resistance and recurrence (7–9). Convincing evidences demonstrated that substantial post-translational heterogeneity exists within cancer cells can affect characteristics of cancer cell stemness (10–12). Therefore, the exploration of intracellular important post-translational regulatory signaling pathways can provide a promising targetable strategy for tumor treatment.

Protein ubiquitination, post-translational modification that regulates all kinds of cellular biological processes, is counteracted upon deubiquitylation by deubiquitinating enzymes (DUBs). DUBs played crucial role in CSC maintenance and differentiation through the regulation of core stem cell transcription factors (SCTFs), such as Oct4, Nanog, and the stem cell surface marker CD133 along with cancer cell sphere formation ability (13–16). In more than one hundred DUBs, ubiquitin-specific proteases (USPs) subfamily is the largest and the most widely studied member. USP13, as an important deubiquitinase member belongs to USPs subfamilies, has been revealed as a potential therapeutic target for its significant role in tumor progression. *USP13* gene is amplified in human lung cancer and clinical samples of non-small cell lung cancer (NSCLC) showed tumor exhibited high USP13 level compared with adjacent normal tissues (17). Accordingly, USP13 depletion attenuated cell proliferation in NSCLC. Moreover, a recent study has shown USP13 was an important target of intrinsic insensitivity to afatinib in EGFR‐mutant NSCLC. Genetic or pharmacological inhibition of USP13 could sensitize EGFR‐mutant NSCLC to EGFR inhibition (18). Although USP13 plays vital role in tumor progression and drug resistance in NSCLC, its precisely biological functions and the regulatory mechanisms in SCLC remain undiscovered.

CSCs are highly reliant on elevated lipogenesis, which is reflected by the upregulation of master enzymes of lipogenesis, such as fatty acid synthase (FASN), ATP-citrate lyase (ACLY) and several fatty acid desaturases, including SCD1 and fatty acid desaturase 1 and 2 (FADS1 and FADS2) (19–22). Previous studies have reported that disorder of lipid metabolism or overactivated lipogenesis pathways are associated with tumor progression and treatment options in SCLC (23, 24). Pharmacological inhibition of lipogenic pathway significantly decreased viability of SCLC cell lines (25). Therefore, targeting lipid metabolism is regarded as a novel strategy against tumor cells, or even CSCs in SCLC.

In this present report, we determined to investigate the contribution of USP13 to SCLC progression. We found ectopic expression of USP13 promotes SCLC stemness and lipogenesis in a FASN-dependent manner, which provides a druggable vulnerability for SCLC patients.

## Materials and methods

### Cell Lines and Cell Culture Conditions

The human small cell lung cancer cell lines NCI-H1048 and NCI-H69, the multidrug-resistant cell line NCI-H69AR, and the human embryonic kidney cell line HEK-293T were purchased from ATCC. H446, H69 and H69AR cells were cultured in RPMI 1640 medium supplemented with 10% fetal bovine serum (FBS, Corning). H1048 cells were cultured in DMEM:F12 (Gibco) supplemented with 10% FBS, 0.005 mg/ml insulin (Sigma), 0.01 mg/ml transferrin (Gibco), 30 nM sodium selenite (Sigma), 10 nM hydrocortisone (Sigma), 10 nM beta-estradiol (Sigma) and 4.5 mM L-glutamine (Gibco). HEK-293T cells were cultured in DMEM (Gibco) supplemented with 10% FBS. Penicillin-streptomycin solution (10,000 U/mL) (Gibco) was added to the prepared culture medium with a 1:100 dilution. Cells were cultured in a humidified incubator at 37°C with 5% CO^2^.

### Lentivirus Packaging and Stable Cell Line Construction

Short hairpin RNA (shRNA) and wild-type plasmids were constructed by SyngenTech company (Beijing). Then, HEK-293T cells were transfected with the recombinant plasmids and packaging plasmids (pLP1, pLP2 and pLP/VSVG; Thermo Fisher Scientific) using Lipofectamine 3000 according to the instructions (Thermo). Forty-eight hours later, lentivirus particles were collected and stored at -80°C. The shRNA sequences used for knockdown were as follows: USP13-shRNA-1 5'-TGATTGAGATGGAGAATAA-3'; USP13-shRNA-2 5'-GCACGAAACTGAAGCCAAT-3'; FASN-shRNA 5'-CCTACTGGATGCGTTCTTCAA-3'.

For stable cell line construction, cells were infected with lentivirus in culture medium supplemented with 5 μg/ml polybrene (Sigma) for 24 hours. The effectively transfected cells were selected with the corresponding antibiotics. The gene expression efficiency was determined by immunoblot analyses.

### RNA Extraction and Quantitative RT–PCR Analysis

Total RNA was extracted using RNA-Quick Purification Kit (ES-RN001, YISHAN Biotechnology) according to the manufacturer’s protocol. Purified RNA was used to generate cDNA with TransScript All-in-One First-Strand cDNA Synthesis SuperMix (TransGen Biotech, AT341-01). qRT–PCR was performed with PerfectStart Green qPCR SuperMix (TransGen Biotech, AQ601-01) on an ABI 7900HT Real-Time PCR Thermocycler (Life Technologies). Relative mRNA expression was determined using the 2^-ΔΔCt^ method, and *actin* was used as an endogenous reference. The following primers were used: *FASN* forward 5′- CAACTCACGCTCCGGAAA-3′, reverse 5′-TGTGGATGCTGTCAAGGG-3′; *actin* forward 5′-ATCAAGATCATTGCTCCTCCTGAG-3′, reverse 5′-CTGCTTGCTGATCCACATCTG-3′.

### Immunoblotting and Immunoprecipitation Assays

Cells were lysed in RIPA buffer (89901, Thermo) containing proteinase and phosphatase inhibitor cocktail (78442, Thermo). Then, the cell lysates were quantified with a BCA protein assay kit (Thermo) according to the manufacturer’s protocol. Equal amounts of protein from cell lysates were separated by SDS-PAGE and performed immunoblotting according previous description (26).

Cells were lysed in IP lysis buffer (87787, Thermo) containing proteinase and phosphatase inhibitor cocktail and rotated at 4 °C for 30 min. After centrifugation, the cell lysates were incubated overnight with indicated antibodies or normal IgG at 4 °C with rotary agitation. Protein A/G agarose beads (sc-2003, Santa Cruz Biotechnology) were added to the lysates and incubated for an additional 3 hours at 4 °C. Beads were washed three times with IP lysis buffer and boiled for 10 min in 1.5% SDS buffer. Whole cell lysates and immunoprecipitates were analyzed by immunoblot assay.

Antibodies that recognize USP13 (ab99421, 1:1000 dilution), Nanog (ab109250, 1:1000 dilution) and Oct4 (ab19857, 1:1000 dilution) were purchased from Abcam. Antibodies against FASN (3180, 1:1000 dilution), HA (3726, 1:1000 dilution), ubiquitin (3933, 1:1000 dilution) and CD133 (64326, 1:1000 dilution) were purchased from CST. Antibody against β-actin (A1978, 1:5000 dilution) was purchased from Sigma. Normal rabbit IgG (2729, 1:5000 dilution) and secondary antibodies, including anti-rabbit IgG HRP-linked antibody (7074, 1:3000 dilution) and anti-mouse IgG HRP-linked antibody (7076, 1:3000 dilution), were purchased from CST.

### Protein Half-Life Detection

For the FASN protein half-life determination, H1048 cells expressing specific plasmids were treated with 100 µg/ml cycloheximide (CHX, HY-12320, MedChem Express) for different periods of time. The cells were collected, and immunoblot analyses were performed with an anti-FASN antibody.

### Immunofluorescence Analysis

H1048 cells were cultured on coverslips and then fixed in 4% paraformaldehyde for 20 min, followed by permeabilization with 0.1% Triton X-100 (in PBS) for 5 min. After washing with PBS, cells were blocked with 5% BSA for 1 hour at room temperature. Cells were then incubated with the indicated primary antibodies at 4°C overnight. After washing with PBS three times, the cells were incubated with secondary antibodies for 1 hour at room temperature and stained with DAPI (Invitrogen) by using ProLong™ Gold Antifade Mountant with DAPI (P36935, Invitrogen). The immunofluorescent staining was observed using a confocal microscope (Olympus).

Antibody that recognizes USP13 (sc-48357, 1:200) was purchased from Santa Cruz Biotechnology. Antibody against FASN (3180, 1:200) was purchased from CST. Anti-mouse IgG (H+L) F(ab’)2 Fragment (Alexa Fluor 488 Conjugate) (4408, 1:1000 dilution) and anti-rabbit IgG (H+L) F(ab’)2 Fragment (Alexa Fluor 555 Conjugate) (4413, 1:1000 dilution) were purchased from CST.

### LC–MS/MS Analysis

USP13 was immunoprecipitated from H1048 cells and separated by SDS–PAGE gel. Proteins in-gel were digested overnight in 12.5 ng/μl trypsin in 25 mM NH4HCO3. The peptides were extracted three times with 60% ACN/0.1% TFA and dried completely in a vacuum centrifuge. LC–MS/MS analysis was performed on a Q Exactive mass spectrometer (Thermo Scientific) coupled to an Easy nLC instrument (Thermo Fisher Scientific) for 60 min.

MS analysis was performed using the MASCOT engine (Matrix Science, London, UK; version 2.2) embedded in Proteome Discoverer 1.4 (Thermo Electron, San Jose, CA.) against the UniProt Human database and the decoy database. MS data were searched against the UniProt database (https://www.uniprot.org/). The cutoff of the global false discovery rate (FDR) for peptide and protein identification was set to 0.01. LC–MS/MS was performed by Shanghai Applied Protein Technology Co., Ltd (Shanghai, China).

### Extreme Limiting Dilution Assay (ELDA)

H1048 or H69 cells were seeded into 96-well ultralow attachment plates (Corning) in DMEM/F12 (Gibco) supplemented with B27 (Gibco), 20 ng/mL epidermal growth factor (Gibco), and 20 ng/mL basic fibroblast growth factor (PeproTech) according previous description (27). After 7 days, the number of positive (sphere formation) wells in each group were uploaded and calculated in the ELDA website (28). The images were observed using an inverted fluorescence microscope (Olympus).

### Measurement of Cellular Cholesterol and Triglyceride Levels

The indicated cells were seeded in 6 cm plates and incubated at 37°C with 5% CO^2^ in an incubator. Until appropriate confluence, cellular cholesterol and triglyceride levels were extracted and determined using Cholesterol Quantitation Kit (MAK043, Sigma) and Triglyceride Quantification Kit (MAK266, Sigma) according to the manufacturer’s protocol, respectively. Cholesterol and triglyceride levels were normalized to the protein concentration.

### CD133^+^ Cells Sorting

H1048 or H69 cells were digested and separated into a single-cell suspension with PBS, then adjusted to a density of 1×10^7^ cells/ml, and subsequently incubated with APC-conjugated anti-CD133 antibody (1:100 dilution) (397906, Biolegend) on ice for 30 min in the dark. Following two washes with PBS, cells were resuspended in 500 µl PBS and subjected to isolation by flow cytometry (BD, LSRII). Negative control was determined by using equal amounts of APC-conjugated immunoglobulin G (IgG) (M1310G05, Biolegend)-stained cells.

### Animal Studies

BALB/c nude mice (4–6 weeks old, female, 14–16 g) were purchased from Beijing HFK Bioscience Co. Ltd. and housed under specific pathogen-free and controlled conditions (25–27, 45–55% humidity, 12 h day/night cycle). The study was approved by the Institutional Animal Care and Use Committee (IACUC) of the National Cancer Center/National Clinical Research Center for Cancer/Cancer Hospital, Chinese Academy of Medical Sciences and Peking Union Medical College, and the methods were carried out in accordance with the approved guidelines.

The cells were subcutaneously injected into the flanks of mice to establish a xenograft tumor. When the size of tumors reached approximately 100 mm^3^, animals were randomly divided into four groups and intraperitoneally injected with 10 mg/kg etoposide (S1225, Selleckchem), or 8 mg/kg TVB-2640 (#S9714, Selleckchem), or etoposide (10 mg/kg) and TVB-2640 (8 mg/kg) combination, or 0.9% saline as control. Tumor length (a) and minor diameter (b) were monitored once a week, and tumor size was calculated using the following formula: volume=a×b^2^/2.

### Patients and Tissue Samples

SCLC samples with paired normal lung tissues were collected from patients who underwent radical resections at the Cancer Hospital of the Chinese Academy of Medical Sciences (Beijing, China) from January 2011 to January 2015. Surgically resected tissues have been pathologically diagnosed and stained with Mayer’s hematoxylin and eosin (HE). After embedding into paraffin, tissue microarray (TMA) was then prepared by Superbiotek, Inc. Ethics approval was granted by the Committee for the Ethics Review of Research Involving Human Subjects of the Cancer Hospital of the Chinese Academy of Medical Sciences. **Table 1** summarized the clinical features of the patients.

**Table 1:** Clinical characteristics of 90 SCLC patients.

Characteristics		Total (cases)	Percentage (%)
Age (years)			
	≤ 60	57	63%
	> 60	33	37%
Gender			
	Male	75	83%
	Female	15	17%
Smoking status			
	Non-smoker	22	24%
	smoker	68	76%
Vascular invasion			
	No	15	17%
	Yes	21	23%
	Unknown	54	60%
Lymph Node Metastasis			
	No	26	≈29%
	Yes	64	≈71%
TNM stage			
	I	24	≈27%
	II	47	≈52%
	III	19	≈21%

### Immunohistochemical (IHC) Analysis

Immunohistochemistry analyses were performed as previously described (29). Briefly, the human SCLC TMA slide was deparaffinized, rehydrated, autoclaved in 10 mM sodium citrate (pH 6.0) for 30 min to unmask antigens, and then incubated with primary antibodies against USP13 (ab99421, 1:200, Abcam), FASN (3180, 1:200, CST), Nanog (ab109250, 1:100, Abcam), or Oct4 (2750, 1:200, CST) at 4°C overnight. The slides were then incubated with secondary antibody, followed by chromogen diaminobenzidine (DAB) staining for signal amplification and detection. The IHC scores were assessed by two independent authors blinded to the treatment groups. IHC scoring was based on the percentage of positive cells and the staining intensity, as previously described (30).

### Oil-Red O Staining

Oil Red O staining was performed on cryosections (6-10 µm) in thickness. In brief, the slides were fixed with formaldehyde, washed with 60% propylene glycerol, and then stained with 0.5% Oil Red O (Sangon Bio) in propylene glycerol for 10 min at 60°C. After staining, the slides were rinsed, counterstained with hematoxylin and mounted in glycerin. The red lipid droplets were visualized by microscopy.

### Public Dataset

Public dataset GSE60052 were downloaded from the Gene Expression Omnibus (GEO, https://www.ncbi.nlm.nih.gov/geo) database.

### Statistical Analysis

Statistical analyses were conducted with a two-tailed unpaired Student’s t test. Each experiment was carried out in at least triplicate, and all data are expressed as the mean ± SD. Kaplan–Meier analysis and log-rank tests were applied for survival analysis (31). The correlation between USP13 and FASN levels was analyzed using a Pearson correlation coefficient. *P* values < 0.05 were considered to be significant. Differences that are statistically significant are labeled with *(*p* < 0.05), **(*p <*0.01), or ***(*p* < 0.001). Statistical analyses were performed by using GraphPad Prism (Version 9).

## Results

### USP13 Is Overexpressed in SCLC and Predicts Poor Clinical Outcomes of SCLC Patients

**Figure 1:**
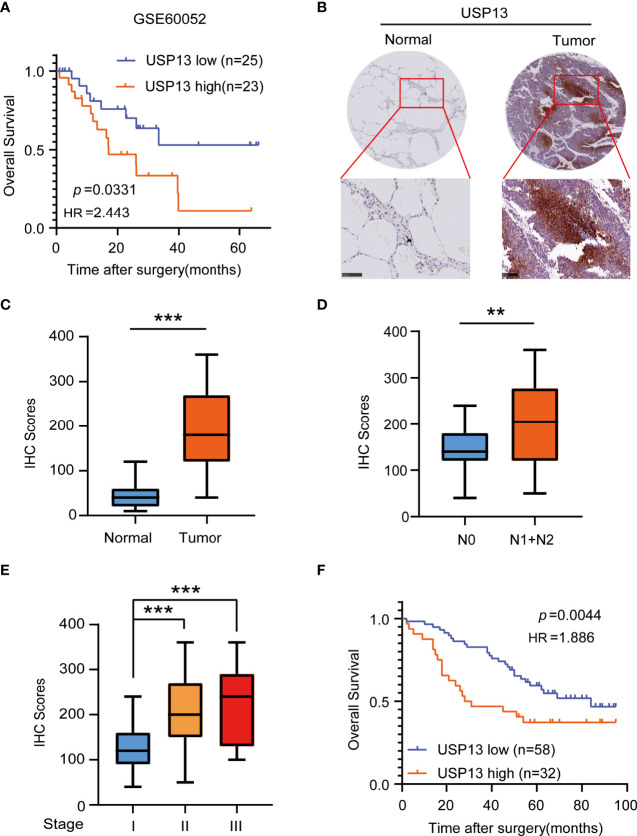
USP13 is overexpressed in SCLC and predicts poor clinical outcomes. **(A)** Kaplan-Meier analysis of overall survival curves from a public dataset (GSE60052) for SCLC patients with low or high USP13 expression. *p* values are calculated using a log-rank test (two-tailed). **(B, C)** Immunohistochemical analysis of USP13 in SCLC tumor specimens (T) and their adjacent normal tissues (N). Representative IHC images are shown (**B**). IHC scores of USP13 expression were calculated (**C**). Scale bars: 500 µm. Data represent the means ± SD of samples. Two-tailed student’s t-test was used. ***p < 0.001. **(D)** The expression of USP13 in SCLC patients with or without lymph node metastasis is shown. Data represent the means ± SD of samples. Two-tailed student’s t-test was used. **p < 0.01. **(E)** USP13 expression levels are shown in SCLC patients of different cancer stages. Data represent the means ± SD of samples. Two-tailed student’s t-test was used. ***p < 0.001. **(F)** The Kaplan–Meier method with a two-tailed log-rank test was used to plot survival curves for SCLC patients with high and low USP13 expression.

To identify the important role of deubiquitinase USP13 in the clinical features of SCLC, we analyzed the mRNA expression of *USP13* in the published profile GSE60052. Overall survival analysis of *USP13* in SCLC patients showed that *USP13* was prognostically detrimental (**Figure 1A**), which indicated further necessary research of USP13. We then collected tumor tissues and the paired peripheral normal lung tissues which were surgically resected from patients diagnosed with SCLC. Immunohistochemical (IHC) analyses revealed that the expression of the USP13 protein was increased in SCLC tissues compared with normal lung tissues (**Figures 1B, C**). Next, we found that USP13 was significantly upregulated in the tissues of SCLC patients with lymph node metastasis (**Figure 1D**). Moreover, compared to patients evaluated as stage I, patients in stage II or stage III overexpressed USP13 (**Figure 1E**). Consistent with the previous analysis, Kaplan–Meier survival analysis demonstrated that high USP13 levels were correlated with poor overall survival of SCLC patients (**Figure 1F**). Collectively, these results demonstrated that USP13 is overexpressed in SCLC and predicts poor clinical outcomes.

### Catalytically Active USP13 Promotes SCLC CSC-like Properties and Rumor Growth

**Figure 2:**
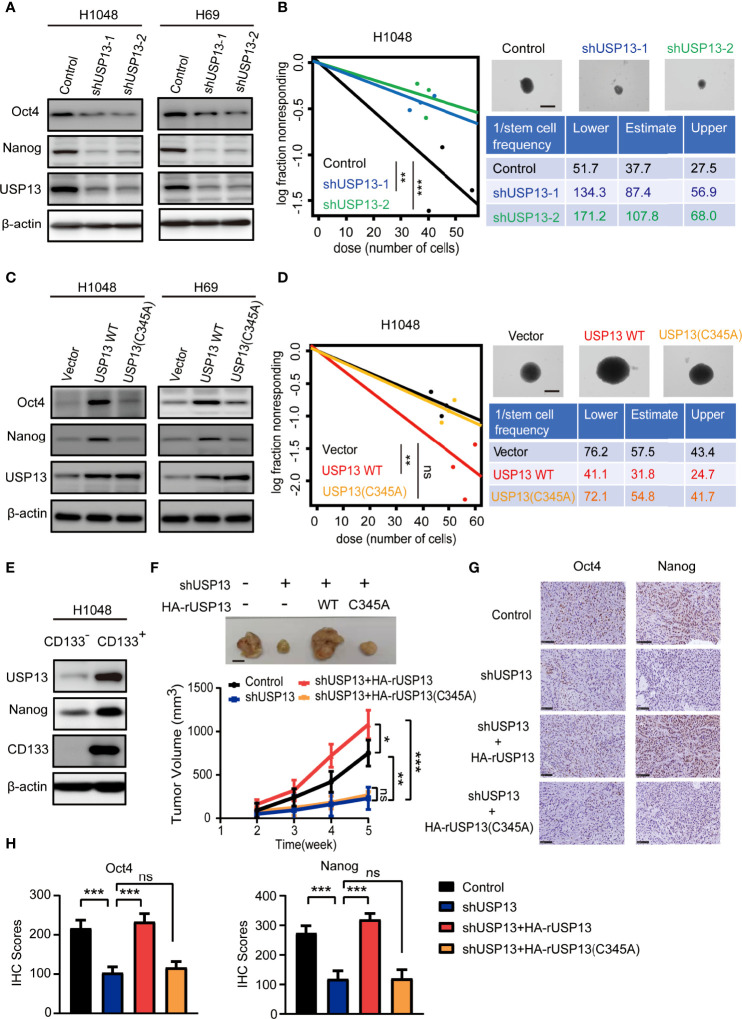
USP13 promotes SCLC CSC-like properties and tumor growth. **(A)** Immunoblot analyses of H1048 (left) and H69 (right) cells with or without USP13 depletion were performed with the indicated antibodies. **(B)** Left: ELDA was performed in H1048 cells with or without USP13 knockdown. Representative sphere images (top right) and stemness frequency illustration of the cells with the upper and lower 95% confidence intervals (bottom right) are shown. Scale bars, 50 μm. Data represent the means ± SD of wells. Two-tailed student’s t-test was used. **p < 0.01, ***p < 0.001. **(C)** Immunoblot analyses of H1048 (left) and H69 (right) cells with or without WT USP13 or catalytically inactive USP13 (C345A) mutant overexpression were performed with the indicated antibodies. **(D)** Left: ELDA was performed in H1048 cells with or without WT USP13 or catalytically inactive USP13 (C345A) mutant overexpression. Representative sphere images (top right) and stemness frequency illustration of the cells with the upper and lower 95% confidence intervals (bottom right) are shown. Scale bars, 50 μm. Data represent the means ± SD of wells. Two-tailed student’s t-test was used. ns, not significant. **p < 0.01. **(E)** CD133^-^ and CD133^+^ subpopulations of H1048 cells were sorted. Immunoblot analyses were performed with the indicated antibodies. **(F)** Tumor formation in immunodeficient mice transplanted with H1048 cells with or without USP13 depletion, or shUSP13 cells combined with reconstituted expression of WT HA-rUSP13 or catalytically inactive HA-rUSP13 (C345A) mutant. Tumor sizes and volumes were measured and calculated (n=5 per group). Data represent the means ± SD of five mice per group. Two-tailed student’s t-test was used. ns, not significant. *p < 0.05, **p < 0.01, ***p < 0.001. **(G, H)** IHC staining of mouse tumor tissues was performed with the indicated antibodies. Representative images are displayed (**G**), and IHC scores were calculated (**H**). Scale bars: 100 μm. Data represent the means ± SD of triplicate samples. Two-tailed student’s t-test was used. ns, not significant. ***p < 0.001.

As CSCs contribute to tumorigenesis, we examined the role of USP13 in SCLC stemness maintenance. The expression of stemness-related factors Oct4 and Nanog was dramatically decreased after depletion of USP13 with two different short hairpin RNAs (shRNAs) in H1048 and H69 cells (**Figure 2A**), which indicated an important role of USP13 in stemness maintenance. Consistently, silencing USP13 significantly inhibited sphere formation ability (**Figure 2B**, **Supplementary Figure 1A**). Conversely, forced expression of WT USP13 but not the catalytically inactive USP13 (C345A) mutant significantly promoted Oct4 and Nanog expression (**Figure 2C**) and sphere formation ability (**Figure 2D**, **Supplementary Figure 1B**). CD133^+^ cells are widely considered to be SCLC stem-like cells (32). To validate the expression levels of USP13 in SCLC CSCs, we enriched CD133^+^ subpopulations from H1048 cells with an anti-CD133 antibody. As shown in **Figure 2E** and **Supplementary Figure 1C**, USP13 expression level in CD133^+^ subpopulation was substantially higher than that in the CD133^-^ subpopulation. Together, these results support a critical role of USP13 which depends on its catalytic function in promoting SCLC stemness.

We next determined the role of USP13 in tumor growth by subcutaneously injecting H1048 cells with or without USP13 depletion, or USP13 depletion combined with WT USP13 or catalytically inactive USP13 (C345A) mutant overexpression (**Supplementary Figure 1D**) in mice. The results showed that mice inoculated with USP13-deficient cells evidently formed smaller tumor masses than those inoculated with control cells, and this reduction was abrogated by reconstituted expression of shRNA-resistant WT USP13 but not the catalytically inactive USP13 (C345A) mutant (**Figure 2F**). Moreover, IHC staining analysis in tumor tissues showed that Nanog and Oct4 expression decreased after USP13 depletion, and this decrease was rescued by reconstituted expression of WT USP13 but not catalytically inactive USP13 (C345A) mutant (**Figures 2G, H**). Hence, our data strongly indicated that USP13 promotes SCLC tumor growth.

### USP13-Dependent FASN Expression Promotes SCLC Stemness and Lipogenesis

**Figure 3:**
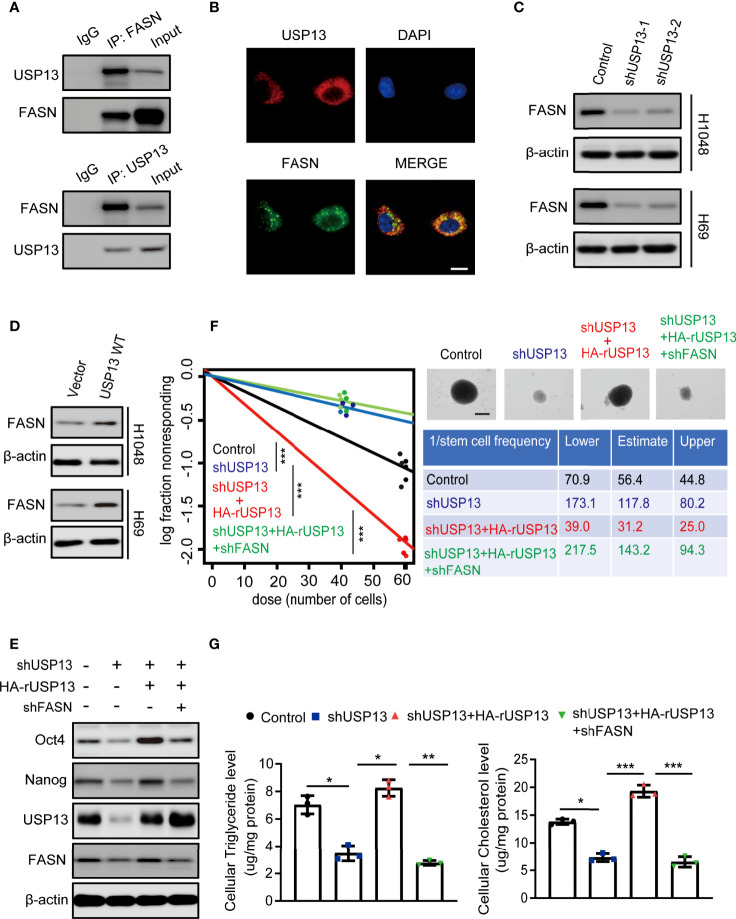
USP13-dependent FASN expression promotes SCLC stemness and lipogenesis. **(A)** Immunoprecipitation and immunoblot analyses were performed with the indicated antibodies in H1048 cells. **(B)** Immunofluorescence analyses were performed with the indicated antibodies in H1048 cells. DAPI was used for nuclear staining. Scale bars: 20 μm. **(C, D)** FASN expression levels were detected in H1048 (top) or H69 (bottom) cells with or without USP13 depletion **(C)** or overexpression **(D)** by immunoblot analyses with an anti-FASN antibody. **(E)** H1048 cells with or without USP13 shRNA expression, or USP13 depletion reconstituted expression of WT HA-rUSP13 with or without FASN depletion were analyzed by immunoblotting with the indicated antibodies. **(F)** Left: ELDA was performed in H1048 cells with or without USP13 shRNA expression, or USP13 depletion combined with reconstituted expression of WT HA-rUSP13 with or without FASN depletion. Representative sphere images (top right) and stemness frequency illustration of the cells with the upper and lower 95% confidence intervals (bottom right) are shown. Scale bars, 50 μm. Data represent the means ± SD of wells. Two-tailed student’s t-test was used. ***p < 0.001. **(G)** Cellular triglyceride levels (left) and cholesterol levels (right) in H1048 cells with or without USP13 shRNA expression, or USP13 depletion combined expression of reconstituted expression of WT HA-rUSP13 with or without FASN depletion were determined. Data shown are the mean ± S.D. (*n*=3). Two-tailed student’s t-test was used. *p < 0.05, **p < 0.01, ***p < 0.001.

To verify mechanisms involved in USP13 regulated SCLC stemness, immunoprecipitation (IP) was performed with an anti-USP13 antibody and mass spectrometry (MS) were used to identify proteins that interacts with USP13. MS analysis revealed 2 unique peptides identical to FASN (**Supplementary Figure 2A**), which is a critical enzyme for the synthesis of palmitate (precursor of cholesterol and triglyceride) from acetyl-CoA and malonyl-CoA and contributes to CSC maintenance (33). We confirmed the interaction between endogenous USP13 and FASN by the co-immunoprecipitation (Co-IP) of H1048 cell lysates (**Figure 3A**). Consistently, USP13 and FASN were shown to interact physically by immunofluorescence (IF) analyses (**Figure 3B**). To determine the importance of USP13 in the regulation of FASN, we then analyzed FASN expression with or without USP13 depletion or overexpression. As shown in **Figures 3C, D**, USP13 downregulation decreased (**Figure 3C**), whereas USP13 overexpression increased FASN protein levels (**Figure 3D**) without affecting FASN mRNA levels (**Supplementary Figure 2B**). To detect the role of USP13-FASN axis in regulating cancer stemness and lipogenesis in SCLC, we stably depleted FASN after WT USP13 reconstitution in USP13 knockdown cells. USP13 depletion in SCLC cells decreased the expression levels of Oct4 and Nanog (**Figure 3E**), reduced the sphere formation ability (**Figure 3F**) and cellular cholesterol and triglyceride levels (**Figure 3G**). In contrast, overexpression of reconstituted WT USP13 in SCLC increased Oct4 and Nanog expression (**Figure 3E**), promoted sphere formation ability (**Figure 3F**) and increased cellular cholesterol and triglyceride levels (**Figure 3G**). Importantly, the effects of USP13 on SCLC cancer stemness maintenance and lipogenesis were abrogated by FASN depletion (**Figures 3E–G**). In addition, IHC analysis and oil red O staining of tumor tissues indicated that USP13-dependent FASN expression increases lipogenesis (**Supplementary Figures 2C, D**). These results indicated USP13 promotes cancer stemness and lipogenesis mainly through FASN.

### USP13 Inhibits Polyubiquitylation-Dependent FASN Degradation

**Figure 4:**
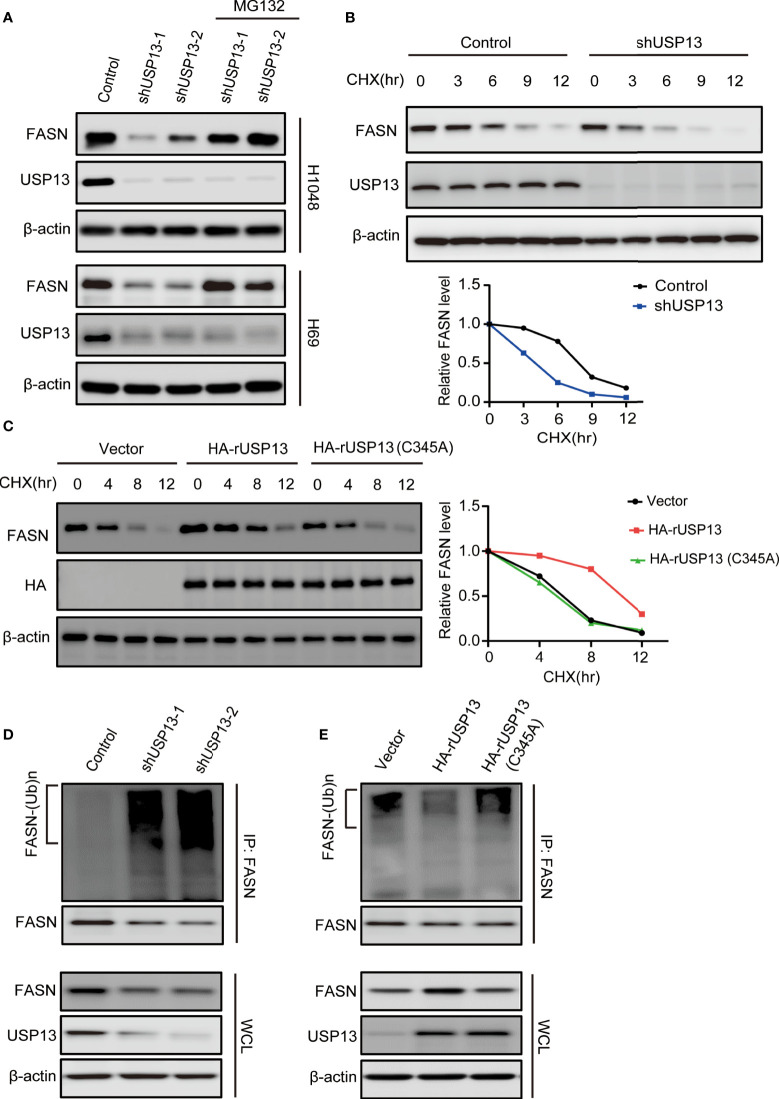
USP13 inhibits polyubiquitylation-dependent FASN degradation. **(A)** H1048 (upper) and H69 (lower) cells expressing two different USP13 shRNAs were treated with MG132 (50 μM) for 8 hours. Immunoblot analyses were performed with the indicated antibodies. **(B)** H1048 cells with or without USP13 depletion were treated with CHX (100 µg/ml) for the indicated periods of time. FASN expression levels were analyzed with an anti-FASN antibody (upper) and quantification of FASN levels relative to β-actin expression levels (lower) were performed. **(C)** H1048 cells with or without WT USP13 or catalytically inactive USP13 (C345A) mutant overexpression were treated with CHX (100 µg/ml) for the indicated periods of time. Immunoblot analyses were performed with the indicated antibodies (left). Quantification of FASN expression levels relative to β-actin expression levels is shown (right). **(D)** H1048 cells expressing two different USP13 shRNAs or a control shRNA were treated with MG132 (50 μM) for 8 hours. Immunoprecipitation and immunoblot analyses were performed with the indicated antibodies. **(E)** H1048 cells with or without WT USP13 or catalytically inactive USP13 (C345A) mutant overexpression were treated with MG132 (50 μM) for 8 hours. Immunoprecipitation and immunoblot analyses were performed with the indicated antibodies.

As a deubiquitinase, we hypothesized that endogenous USP13 could regulate FASN stability. We treated USP13 knockdown cells with the proteasome inhibitor MG-132 and found that inhibition of FASN expression by USP13 depletion was clearly blocked by MG-132 treatment in H1048 and H69 cells (**Figure 4A**). To determine whether USP13 stabilizes the FASN protein through deubiquitination, we first examined FASN protein turnover by using cycloheximide (CHX) and traced the protein levels. Indeed, the FASN protein was gradually degraded with CHX treatment. As anticipated, the FASN protein half-life was decreased after depletion of USP13 (**Figure 4B**), while ectopic WT USP13 but not catalytically inactive USP13 (C345A) mutant expression largely increased FASN stability (**Figure 4C**). Moreover, we found that depletion of USP13 enhanced the ubiquitination level of endogenous FASN (**Figure 4D**). In contrast, forced expression of WT USP13 but not the catalytically inactive USP13 (C345A) mutant reduced the ubiquitination of FASN (**Figure 4E**). Overall, our experiments indicate that USP13 promotes FASN protein stability by decreasing polyubiquitination of FASN and thus prevents its degradation through the proteasome pathway.

### FASN is Positively Correlates with USP13

**Figure 5:**
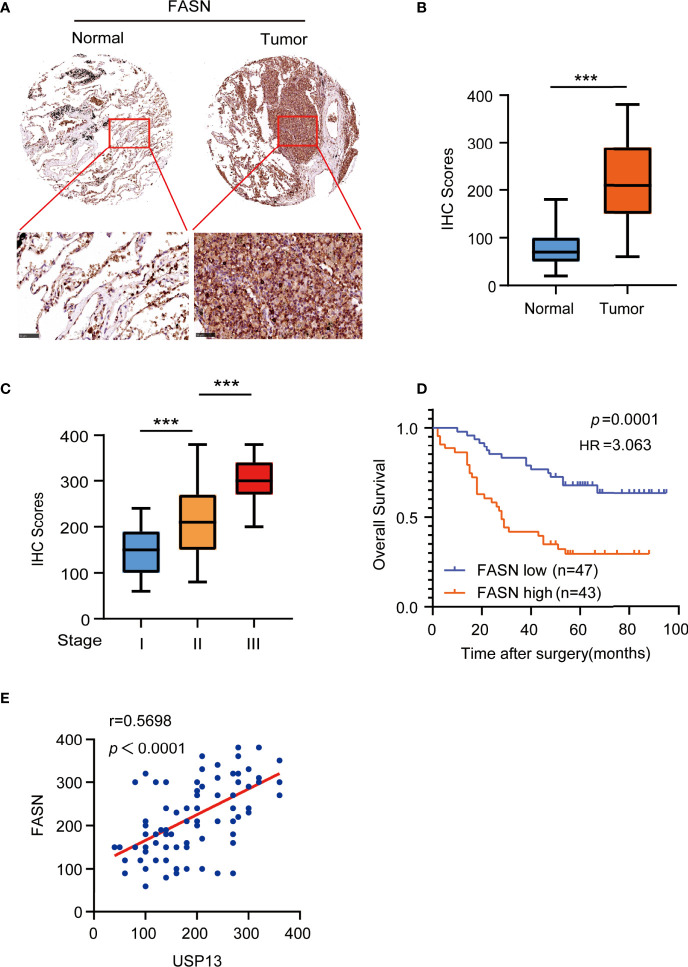
FASN is positively correlates with USP13. **(A, B)** Representative images **(A)** of IHC staining for FASN protein on tissue microarray (TMA) composed of SCLC tumor specimens (T) with their adjacent normal tissues (N). IHC score **(B)** was calculated. Scale bars: 100 μm. Data represent the means ± SD of samples. Two-tailed student’s t-test was used. ***p < 0.001. **(C)** FASN expression levels are shown in SCLC patients of different cancer stages. Data represent the means ± SD of samples. Two-tailed student’s t-test was used. ***p < 0.001. **(D)** Kaplan-Meier plots of the overall survival time of 90 SCLC patients with low or high expression levels of FASN. *p* values are calculated using a log-rank test (two-tailed). **(E)** The correlation between USP13 and FASN expression in an SCLC tissue microarray was analyzed using a two-tailed Pearson correlation coefficient.

To determine the clinical significance of FASN expression, we performed IHC analyses of SCLC tissue specimens. IHC staining showed FASN was highly upregulated in SCLC tissues than in the paired adjacent normal tissues (**Figures 5A, B**). More importantly, the expression of FASN was stage-dependent (**Figure 5C**). In addition, patients with high FASN expression had shorter survival duration than those with low FASN expression (**Figure 5D**). Consistent with previous results, FASN protein expression levels were positively correlated with USP13 expression levels (**Figure 5E**). These results strongly suggested that USP13-stabilized FASN expression promotes clinical aggressiveness of SCLC.

### FASN Inhibition Attenuates SCLC Lipogenesis, Self-Renewal Properties, Chemotherapy Resistance and USP13-Dependent Tumorigenesis

**Figure 6:**
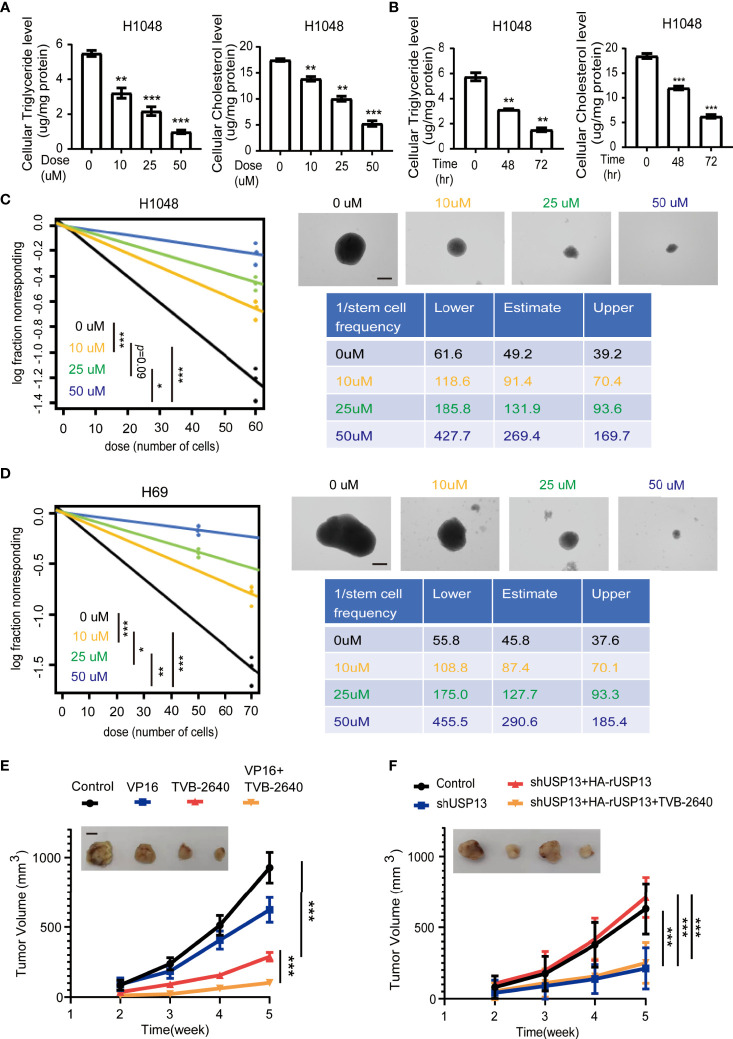
FASN inhibition attenuates SCLC lipogenesis, self-renewal properties, chemotherapy resistance and USP13-dependent tumorigenesis. **(A)** Triglyceride (left) and cholesterol (right) levels were measured in H1048 cells treated with the FASN inhibitor TVB-2640 at the indicated doses for 72 hours. DMSO was used as the vehicle control. Data represent the means ± SD of triplicate samples. Two-tailed student’s t-test was used. **p < 0.01, ***p < 0.001. **(B)** Triglyceride (left) and cholesterol (right) levels were measured in H1048 cells after 50 μM FASN inhibitor TVB-2640 treatment for the indicated periods of time. Data represent the means ± SD of triplicate samples. Two-tailed student’s t-test was used. **p < 0.01, ***p < 0.001. **(C, D)** Left: ELDA was performed in H1048 **(C)** and H69 **(D)** cells with or without FASN inhibitor TVB-2640 treatment at the indicated dose. Representative sphere images (top right) and stemness frequency illustration of the cells with the upper and lower 95% confidence intervals (bottom right) are shown. Scale bars, 50 μm. Data represent the means ± SD of wells. Two-tailed student’s t-test was used. *p < 0.05, **p < 0.01, ***p < 0.001. **(E)** Nude mice were subcutaneously injected with H69AR cells and intraperitoneally injected with indicated drugs when the size of the tumor reached approximately 100 mm^3^. Tumor sizes and volumes were measured and calculated (n=5 per group). Data represent the means ± SD of five mice per group. Two-tailed student’s t-test was used. *p < 0.05, **p < 0.01, ***p < 0.001. **(F)** H1048 cells with or without USP13 depletion, or shUSP13 cells combined with reconstituted expression of WT HA-rUSP13 were subcutaneously injected into nude mice and then with or without TVB-2640 treatment. Tumor sizes and volumes were measured and calculated (n=5 per group). Data represent the means ± SD of five mice per group. Two-tailed student’s t-test was used. ns, not significant. ***p < 0.001.

Since FASN is a key enzyme involved in USP13-promoted cancer stemness, we further examined whether FASN inhibitor could be a favorable treatment strategy. TVB-2640, a highly potent and selective FASN inhibitor, was designed to reduce hepatic fat in nonalcoholic fatty liver disease (NAFLD) and nonalcoholic steatohepatitis (34, 35). More importantly, with compelling support as an oncology therapeutic drug, TVB-2640 was the first highly selective FASN inhibitor to enter clinical studies (36). We found that TVB-2640 treatment decreased cellular cholesterol and triglyceride levels and reduced sphere formation ability in a dose- and time-dependent manner in both H1048 and H69 cells (**Figures 6A–D**, **Supplementary Figures 3A, B**). Given that FASN expression is associated with chemoresistance (37), targeting lipid metabolism might be a new potential therapy for SCLC patients with acquired drug-resistance. We subcutaneously injected multi-drugs resistant H69AR cells into mice (38), then treated with VP16 (etoposide) or TVB-2640 for monotherapy, or concurrent therapy with VP16 and TVB-2640. Consistent with previous study (38), tumors in the VP16 treatment group displayed slight shrinkage (**Figure 6E**). Importantly, TVB-2640 treatment dramatically suppressed tumor growth (**Figure 6E**). In addition, the group treated with both VP16 and TVB-2640 showed an improved better response than the group treated with TVB-2640 alone (**Figure 6E**), indicated synergistic treatment effect. IHC staining analysis confirmed that lipogenesis inhibition by TVB-2640 repressed SCLC stemness features, reflected by decreased Nanog expression, and in combination with classical chemotherapeutics induced a dramatic synergistic effect (**Supplementary Figure 3C**). To further study whether TVB-2640 treatment can reverse USP13-mediated tumor formation, we subcutaneously injected H1048 cells with or without USP13 depletion, or H1048 USP13-depleted cells with reconstituted expression of shRNA-resistant WT USP13 with or without TVB-2640 treatment. Consistent with previous result, USP13 depletion dramatically suppressed tumor growth, and forced overexpression of WT HA-rUSP13 rescued the decreased tumor volume (**Figure 6F**). Importantly, TVB-2640 treatment can significantly reduce the tumorigenesis caused by USP13 overexpression. Together, these data show that the FASN inhibitor TVB-2640 can be used as a potential chemotherapy combination anti-SCLC drug.

## Discussion

The molecular function of USP13 in tumorigenesis has been controversial in different cancers according to previous studies. USP13 was first found to deubiquitinate tumor suppressor protein PTEN in human breast cancer cells, which indicated a tumor-suppressing role for USP13 (39). However, further studies revealed that USP13 may have context-dependent functions in cancer development by interacting with different substrates to regulate protein stability. *USP13* gene is amplified in serious ovarian cancers and specifically deubiquitinates and thus upregulates two key metabolic key enzymes, ATP citrate lyase (ACLY) and oxoglutarate dehydrogenase (OGDH). As a consequence, USP13 overexpression is correlated with poor clinical outcome (40). In lung and ovarian cancer cells, USP13 deubiquitinates and stabilizes MCL1, a key member of the anti-apoptotic BCL-2 family. Pharmacological inhibition of USP13 with spautin-1 significantly inhibits tumor growth and increases tumor cell sensitivity to BH3 mimetic inhibitors, which suggests that targeting USP13 may be a valuable strategy for cancer treatment (41). As a highly expressed protein in glioma stem cells (GSCs), USP13 maintains GSC self-renewal abilities by stabilizing the critical transcription factor c-Myc (42). Consistently, we found that USP13 plays an oncogenic role to maintain SCLC stemness and tumorigenic potential, which is dependent on its catalytic activity. Importantly, USP13 expression is positively correlated with cancer progression and predicts poor survival of SCLC patients. Further mechanistic studies revealed that USP13 interacts with and stabilizes FASN by reducing FASN polyubiquitination, suggesting a putative target for SCLC treatment.

Previous studies have shown that cancer cells reprogram lipogenic metabolism in response to the massive demand for macromolecules and bioenergy (43, 44), and that increased expression of FASN is a prominent feature (45). In KRAS signaling active lung adenocarcinomas, FASN is a primary responder that induces elevated lipogenesis which mediated by the ERK pathway. Inhibition of FASN by cerulenin blocked proliferation of KRAS-driven lung cancer cells, indicating a promising role of lipid metabolism in tumor treatment (46). Subsequent studies in EGFR mutated non‐small cell lung cancer confirmed that FASN-associated fatty acid metabolic pathway upregulation was the main principal for tyrosine kinase inhibitor (TKI)‐resistant EGFR mutated NSCLC growth (47). In our studies, FASN was induced by the stemness-related deubiquitinase USP13 in SCLC, implying that lipogenesis can augment the self-renewal property, and that effectively inhibiting FASN activity may provide an alternative treatment. Although FASN inhibitors, including C75, C93, GSK837149A, Orlistat and TVB-3166, have demonstrated preclinical antitumor activity in cancer cell lines and xenograft models (45, 48), none of these compounds have been tested in cancer patients due to side effects. Therefore, we selected the FASN inhibitor TVB-2640, which has a favorable tolerability profile and entered a phase II clinical trial (36), as treatment strategy for SCLC. Our results showed that accompanied by decreased triglyceride and cholesterol levels, TVB-2640 significantly suppressed self-renewal ability of SCLC. Further *in vivo* animal studies revealed TVB-2640 sensitized chemotherapy-resistant tumor cells to etoposide treatment and inhibited USP13-dependent cancer stemness and tumor growth, suggesting a crucial role of lipogenic pathway in SCLC proliferation and drug resistance. Previous research has identified that MEK5/ERK5 dual kinase axis supporting SCLC survival heavily relies on the mevalonate pathway, which controls cholesterol synthesis (25). Corroborative evidence has shown that mutated FASN, which may affect dimer formation or enzyme activity, occurs in SCLC patients and indicates a better prognosis in those who have received chemotherapy (49). These results support that abnormal expression of proteins involved in lipogenic pathways synergistically promotes cancer cell survival, which provides a more efficacious strategy for treatment of SCLC. Finally, the clinical significance of FASN expression in our cohort was evidenced by its positive association with SCLC patient clinical stage and poor overall survival.

## Conclusions

In this study, we showed that USP13 promotes SCLC stemness and lipogenesis by inhibiting proteasome-dependent FASN degradation. Pharmacological inhibition of FASN with the small molecule TVB-2640 impaired self-renewal and tumor growth ability of SCLC.

## Data Availability Statement

The datasets presented in this study can be found in online repositories. The names of the repository/repositories and accession number(s) can be found below: http://proteomecentral.proteomexchange.org/, PXD032993.

## Ethics Statement

The study was approved by the Institutional Animal Care and Use Committee (IACUC) of the National Cancer Center/National Clinical Research Center for Cancer/Cancer Hospital, Chinese Academy of Medical Sciences and Peking Union Medical College, and the methods were carried out in accordance with the approved guidelines. Written informed consent was obtained from the individual(s) for the publication of any potentially identifiable images or data included in this article.

## Author Contributions

YG and JW conceived and designed the study; JH provided critical scientific input. JW, WL, RL, HC, SS, FS, and YY performed the experiments. JH, XF, SG, and YG established the patient cohort and biobanking of specimen for tissue microarray. JW wrote the draft manuscript. YG revised the manuscript. All authors contributed to the article and approved the submitted version.

## Funding

This work was supported by National Key R&D Program of China (2020AAA0109500, JH), National Natural Science Foundation of China (82122053, YG, 82188102, JH), the Beijing Municipal Science & Technology Commission (Z191100006619118, JH), R&D Program of Beijing Municipal Education commission (KJZD20191002302, JH), CAMS Initiative for Innovative Medicine (2021-1-I2M-012, JH, 2021-1-I2M-015, SG), Non-profit Central Research Institute Fund of Chinese Academy of Medical Sciences (2021-PT310-001, YG), and Aiyou Foundation (KY201701, JH).

## Conflict of Interest

The authors declare that the research was conducted in the absence of any commercial or financial relationships that could be construed as a potential conflict of interest.

## Publisher’s Note

All claims expressed in this article are solely those of the authors and do not necessarily represent those of their affiliated organizations, or those of the publisher, the editors and the reviewers. Any product that may be evaluated in this article, or claim that may be made by its manufacturer, is not guaranteed or endorsed by the publisher.

## Supplementary Material

Supplemental Figure 1 **USP13 promotes SCLC CSC-like properties: (A)** Left: ELDA was performed in H69 cells with or without USP13 knockdown. Representative sphere images (top right) and stemness frequency illustration of the cells with the upper and lower 95% confidence intervals (bottom right) are shown. Scale bars, 50 μm. Data represent the means ± SD of wells. Two-tailed student’s t-test was used. ***p < 0.001. **(B)** Left: ELDA was performed in H69 cells with or without WT USP13 or catalytically inactive USP13 (C345A) mutant overexpression. Representative sphere images (top right) and stemness frequency illustration of the cells with the upper and lower 95% confidence intervals (bottom right) are shown. Scale bars, 50 μm. Data represent the means ± SD of wells. Two-tailed student’s t-test was used. ns, not significant. ***p < 0.001. **(C) **CD133^-^ and CD133^+^ subpopulations of H69 cells were sorted. Representative cell fractions were separated by flow cytometry sorting (upper). Immunoblott analyses were performed with the indicated antibodies (lower). **(D)** Immunoblot analyses were performed in H1048 cells with or without USP13 depletion, or shUSP13 cells combined with reconstituted expression of WT HA-rUSP13 or catalytically inactive HA-rUSP13 (C345A) mutant the indicated antibodies.

Supplemental Figure 2 **USP13-dependent FASN expression promotes SCLC stemness and lipogenesis: (A)** H1048 cells were immunoprecipitated with anti-USP13 antibody and analyzed by mass spectrometry. The selected FASN peptide identified by mass spectrometry analyses was shown. **(B)** Relative mRNA level of FASN was detected in H1048 cells with or without USP13 depletion (top) or overexpression (bottom). Data shown are the mean ± S.D. (*n*=3). ns, not significant.**(C, D)** IHC staining of FASN with anti-FASN antibody and the oil red O staining were performed by using tumor tissues as described in Figure 2F. Representative images are displayed **(C)**. IHC scores (**D**, top right) and oil red O-stained areas (**D**, bottom right) were calculated. Scale bars: 100 μm. Data represent means ± SD of triplicate samples. Two-tailed student’s t-test was used. ns, not significant. *p < 0.05, **p < 0.01, ***p < 0.001.

Supplemental Figure 3 **FASN inhibition attenuates SCLC lipogenesis: (A)** Triglyceride (left) and cholesterol (right) levels were measured in H69 cells based on FASN inhibitor TVB-2640 treatment with indicated dose for 72 hours. DMSO was used as control vehicle. Data represent means ± SD of triplicate samples. Two-tailed student’s t-test was used. **p < 0.01, ***p < 0.001. **(B)** Triglyceride (left) and cholesterol (right) levels were measured in H69 cells after 50uM FASN inhibitor TVB-2640 treatment for the indicated periods of time. Data represent means ± SD of triplicate samples. Two-tailed student’s t-test was used. ***p < 0.001. **(C)** IHC staining of Nanog expression and the oil red O staining were performed with mouse tumor tissues. Representative images are displayed (left). IHC scores (top right) and oil red O-stained areas (bottom right) were calculated. Scale bars: 100 μm. Data represent the means ± SD of triplicate samples. Two-tailed student’s t-test was used. ns, not significant. *p < 0.05, **p < 0.01.

## Abbreviations

SCLC, Small-cell lung cancer; CSC, Cancer stem-like cell; USP13, Ubiquitin-specific peptidase 13; FASN, Fatty acid synthase; TMA, Paraffin-embedded tissue microarray; HR, hazard ratio; shRNA, Short hairpin RNA; DUB, Deubiquitinating-enzyme; Ub, Ubiquitin; USPs, Ubiquitin-specific proteases; WT, Wild type; IP, Immunoprecipitation; CHX, Cycloheximide; IHC, Immunohistochemistry; qRT-PCR, Real-time quantitative reverse transcriptase-polymerase chain reaction; MS, Mass spectrometry; OS, Overall survival.
