# Supplementary figures and images for: The Deubiquitinase USP13 Maintains Cancer Cell Stemness by Promoting FASN Stability in Small Cell Lung Cancer
Source: Front Oncol. 2022 Jul 11;12:899987. doi: 10.3389/fonc.2022.899987 (PMC9309731; doi:10.3389/fonc.2022.899987)

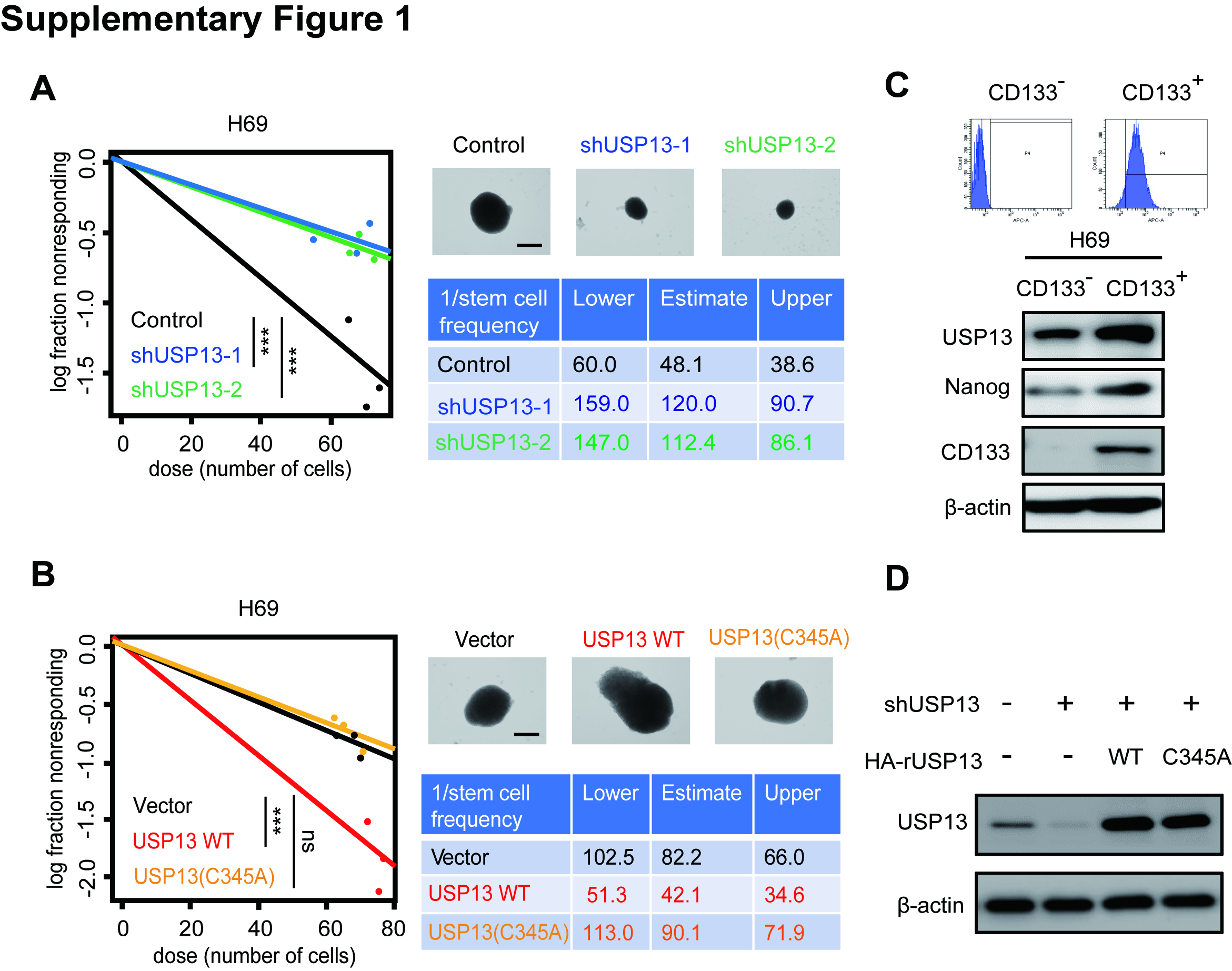

Supplement: Supplemental Figure 1 — USP13 promotes SCLC CSC-like properties: (A) Left: ELDA was performed in H69 cells with or without USP13 knockdown. Representative sphere images (top right) and stemness frequency illustration of the cells with the upper and lower 95% confidence intervals (bottom right) are shown. Scale bars, 50 μm. Data represent the means ± SD of wells. Two-tailed student’s t-test was used. ***p < 0.001. (B) Left: ELDA was performed in H69 cells with or without WT USP13 or catalytically inactive USP13 (C345A) mutant overexpression. Representative sphere images (top right) and stemness frequency illustration of the cells with the upper and lower 95% confidence intervals (bottom right) are shown. Scale bars, 50 μm. Data represent the means ± SD of wells. Two-tailed student’s t-test was used. ns, not significant. ***p < 0.001. (C) CD133- and CD133+ subpopulations of H69 cells were sorted. Representative cell fractions were separated by flow cytometry sorting (upper). Immunoblott analyses were performed with the indicated antibodies (lower). (D) Immunoblot analyses were performed in H1048 cells with or without USP13 depletion, or shUSP13 cells combined with reconstituted expression of WT HA-rUSP13 or catalytically inactive HA-rUSP13 (C345A) mutant the indicated antibodies. [file Image_1.tif]

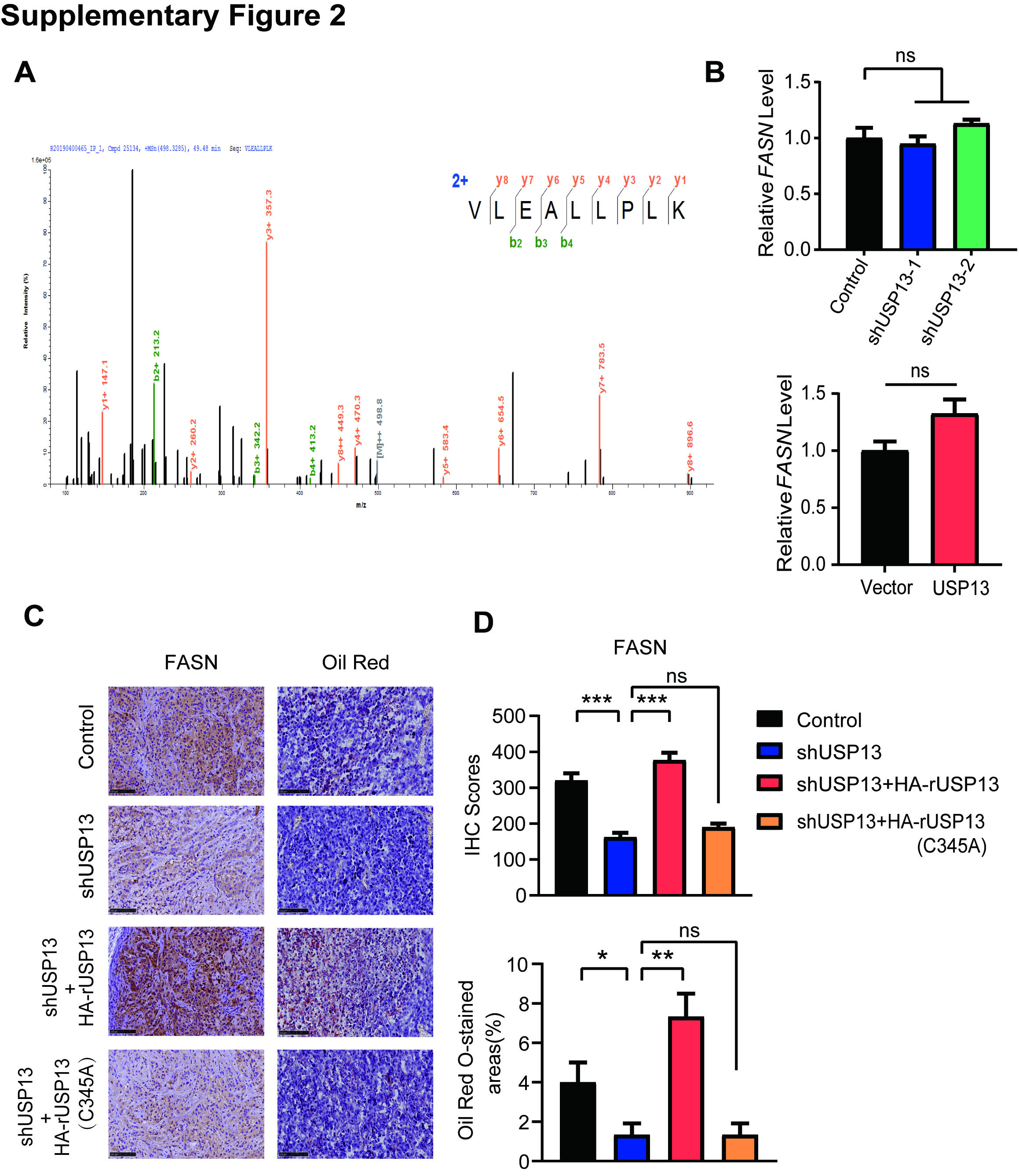

Supplement: Supplemental Figure 2 — USP13-dependent FASN expression promotes SCLC stemness and lipogenesis: (A) H1048 cells were immunoprecipitated with anti-USP13 antibody and analyzed by mass spectrometry. The selected FASN peptide identified by mass spectrometry analyses was shown. (B) Relative mRNA level of FASN was detected in H1048 cells with or without USP13 depletion (top) or overexpression (bottom). Data shown are the mean ± S.D. (n=3). ns, not significant.(C, D) IHC staining of FASN with anti-FASN antibody and the oil red O staining were performed by using tumor tissues as described in Figure 2F. Representative images are displayed (C). IHC scores (D, top right) and oil red O-stained areas (D, bottom right) were calculated. Scale bars: 100 μm. Data represent means ± SD of triplicate samples. Two-tailed student’s t-test was used. ns, not significant. *p < 0.05, **p < 0.01, ***p < 0.001. [file Image_2.tif]

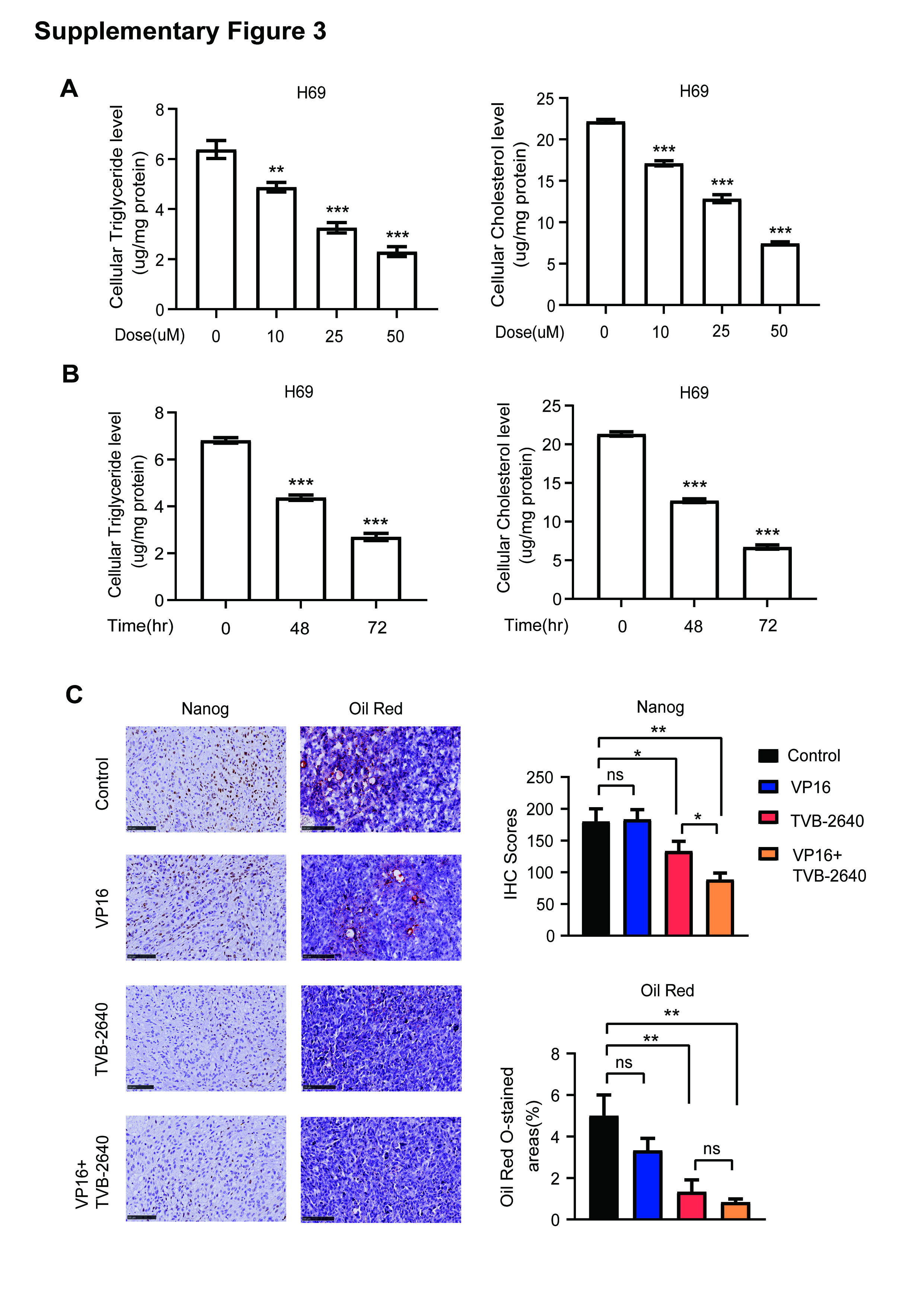

Supplement: Supplemental Figure 3 — FASN inhibition attenuates SCLC lipogenesis: (A) Triglyceride (left) and cholesterol (right) levels were measured in H69 cells based on FASN inhibitor TVB-2640 treatment with indicated dose for 72 hours. DMSO was used as control vehicle. Data represent means ± SD of triplicate samples. Two-tailed student’s t-test was used. **p < 0.01, ***p < 0.001. (B) Triglyceride (left) and cholesterol (right) levels were measured in H69 cells after 50uM FASN inhibitor TVB-2640 treatment for the indicated periods of time. Data represent means ± SD of triplicate samples. Two-tailed student’s t-test was used. ***p < 0.001. (C) IHC staining of Nanog expression and the oil red O staining were performed with mouse tumor tissues. Representative images are displayed (left). IHC scores (top right) and oil red O-stained areas (bottom right) were calculated. Scale bars: 100 μm. Data represent the means ± SD of triplicate samples. Two-tailed student’s t-test was used. ns, not significant. *p < 0.05, **p < 0.01. [file Image_3.tif]
